# The Management and Clinical Outcomes of Pregnancy in a Female With Glycogen Storage Disease Type IIIA Caused by Rare Variant

**DOI:** 10.1002/jmd2.70030

**Published:** 2025-07-02

**Authors:** Nuria Puente‐Ruiz, Saru Palaniappan, Alison Woodall, Robert Cooper, Allyson Terry, Andrew Oldham, Abigail Rousseau, Christopher Campbell, Pradeep Vasudevan, Karolina M. Stepien

**Affiliations:** ^1^ Adult Inherited Metabolic Diseases, Salford Royal NHS Foundation Trust Salford UK; ^2^ Department of Internal Medicine Marqués de Valdecilla University Hospital. Instituto de Investigación Valdecilla (IDIVAL). Centro de Investigación Biomédica en Red en Enfermedades Raras (CIBERER) Santander Spain; ^3^ Obstetrics and Gynaecology Department Whiston Hospital Warrington UK; ^4^ Cardiology Department Liverpool Heart and Chest Hospital Liverpool UK; ^5^ Department of Nutrition and Dietetics Alder hey Children's NHS Foundation Trust Liverpool UK; ^6^ Manchester Centre for Genomic Medicine, Saint Mary's Hospital Manchester University NHS Foundation Trust Manchester UK; ^7^ Clinical Genetics Department University Hospitals Leicester NHS Trust Leicester UK; ^8^ Division of Cardiovascular Sciences University of Manchester Manchester UK

**Keywords:** cardiac MR, cardiomyopathy, genomic rearrangement, glycogen storage disease type IIIA, pregnancy

## Abstract

Glycogen storage disease type III (GSD III) is an inborn error of carbohydrate metabolism with an autosomal recessive inheritance pattern. Phenotypically, patients can manifest a broad clinical spectrum. Most patients affected with GSD IIIA (85%) have a non‐functional GDE enzyme primarily affecting the liver and cardiac/skeletal muscle (Type IIIA). Initial clinical manifestations of GSD IIIA present in the first year of life. Presentation is very similar to GSD type I. Up to 98% of children affected have hepatomegaly, hypoglycaemia (53%) with marked ketosis (34%), short stature (49%), delayed puberty, and frequent infections (17%). In adulthood, they may have cirrhosis, adenomas, or hepatocarcinomas (11%), cardiomyopathy (58%) and myopathy (34%). Pregnancy has been documented in women with GSD III. Nutritional requirements are increased during pregnancy, especially in the third trimester. We report the management of a woman with GSD IIIA found to be compound heterozygous for two pathogenic AGL variants, c.798C>G p.(Tyr266Ter) and c.4258_4259ins? p.(Asp1420fs), who had a planned pregnancy. Cardiac outcomes are also described/discussed.

**Summary**

- Women with GSD IIIA are at risk of cardiac complications and require close surveillance of cardiology specialist throughout the peripartum period.
- Women with GSD III are at risk of alterations in the metabolic rate during pregnancy and the post‐partum period.
- Close management of diet with regular monitoring is necessary to ensure that the risk of hypoglycaemia is minimised.
- Post‐partum monitoring is essential for the patients to fully manage direct and indirect complications for pregnancy in GSD III.

## 1 Introduction

Glycogen storage disease type III (GSDIII) (OMIM#232400) is an inborn error of carbohydrate metabolism with an autosomal recessive inheritance pattern. It is caused by biallelic mutations in the *AGL* gene located on chromosome 1p21, which codes for amylo‐1,6‐glucosidase or debranching enzyme (GDE) (EC 3.2.1.33). GDE metabolises branch points of glycogen to produce glucose for glucose homeostasis. GDE deficiency results in the storage of abnormal glycogen with short outer chains stored in the liver, heart, blood cells, and muscle [1, 2].

GSD III has an estimated incidence of 1 in 100 000 live births. The highest rates have been reported in Inuit population in Nunavik, Canada (1 in 2500 births), Faroe Islands (1 in 3600 births) or in North African Jews (1 in 5400 births) [3, 4, 5].

Phenotypically, patients can manifest a wide clinical spectrum. Most patients (85%) have a non‐functional GDE enzyme affecting the liver and cardiac/skeletal muscle (Type IIIA). For approximately 15% of patients, GDE enzyme deficiency affects the liver only (Type IIIB) [2, 6, 7]. Additional extremely rare cases have presented with a selective loss of a glucosidase activity (Type IIIC) involving muscle only, or transferase activity (Type IIID) which affects muscle and liver [8, 9].

The first clinical manifestations of GSD IIIA present in the first year of life and closely resemble GSD type I. Up to 98% of children affected have hepatomegaly, hypoglycaemia (53%) with marked ketosis (34%), short stature (49%), delayed puberty, and frequent infections (17%). In adulthood, they may have cirrhosis, adenomas, or hepatocarcinomas (11%), and myopathy (34%) [6, 10]. GSD III is a highly heterogeneous condition with 58% of patients developing decompensated structural cardiomyopathy around the time of increased glucose requirements (childhood) [6].

There is no cure for GSD IIIA. One of management's key goals is to prevent symptoms through dietary management. Children with GSD IIIA are advised to have a high protein intake (± 3‐4 g/kg/day). A regular intake of carbohydrate foods, every 3–4 h, and uncooked cornstarch is advised to prevent hypoglycaemia. Additional nocturnal enteral feeding is also often recommended to further prevent hypoglycaemia. Adults are advised to follow a high‐protein diet and a regular eating pattern, including regular carbohydrates. Uncooked cornstarch is also taken by some adults with GSD IIIA as a further measure to prevent hypoglycaemia [6, 11, 12].

Nutritional requirements are increased during pregnancy, especially in the second and third trimesters. There are a few cases described in the literature outlining the principles of management during pregnancy and labour [11, 12, 13, 14, 15]. We report the management of a case of a pregnant woman with GSD IIIA, including cardiac outcomes.

## 2 Case

Our patient was born at term without complications. The clinical and biochemical diagnosis of GSD type III A was confirmed by liver biopsy at the age of 2.5 years. At diagnosis neither hypoglycaemia nor myopathy was apparent.

During the first decade of life creatinine kinase (CK) concentration remained stable at around 1600 U/L. By the age 17, she was exercising 5 days a week, which resulted in CK rising to 3824 U/L.

At the age of 19, she transitioned to adult metabolic service. She experienced a few episodes of hypoglycaemia without requiring an emergency regimen. She reported a few episodes of muscle weakness, shaking, and pain in her lower limbs towards the end of the day.

Physical examination revealed a high BMI (40.7 kg/m^2^). Routine investigations showed blood glucose concentration and urine ketones were negative. CK concentration fluctuated between 2227 U/L and 3763 U/L. Folic acid and vitamin D were at the lower end of the normal range. Liver function tests showed raised ALT at 169 U/L prior to pregnancy; lipid profile showed total cholesterol 5.5 mmol/L and serum triglycerides 3.7 mmol/L. Echocardiogram showed mild left ventricular hypertrophy (IVSd 13 mm) and mild mitral regurgitation.

Physiotherapy assessment showed that she was independently mobile; her muscle strength testing revealed normal muscle power in her upper and lower limbs. She was advised to reduce her physical activity below 70% level of intensity with the aim of minimising muscle symptoms.

Assessment for fasting tolerance showed that her maximum fasting time was 9 h. Her diet showed a regular intake of carbohydrates, including 25 g of cornstarch each night at 1 am. Her protein intake was less than recommended at approximately 13% of total energy intake, and her calcium intake did not meet basic requirements. Advice was provided to increase her protein intake to 25% of total energy intake, aiming for 120 g of protein daily. She was also encouraged to take folic acid supplements, cholecalciferol 40 000 units, low glycaemic index diet and medium‐chain triglyceride (MCT) oil/Procal.

## 3 Antenatal Management

At the age of 24, she became pregnant. Both her raised BMI at 43 kg/m^2^ and previous history of smoking were risk factors for pre‐existing cardiomyopathy. She worked as a health care assistant in a care home on long 12‐h shifts. She had hypoglycaemia awareness and self‐managed her symptoms with diet. She did not report dyspnoea, orthopnoea, or palpitations. During the 1st trimester, she suffered from morning sickness but did not use her emergency regimen. She suffered a viral infection in the first trimester of her pregnancy but did not require hospital admission.

Baseline echo performed at 16 weeks of pregnancy reported mild concentric left ventricular hypertrophy. Her obstetrician prescribed aspirin 150 mg/day, folic acid 5 mg daily, and vitamin D 400 IU/day. Due to her venous thromboembolism risk score of 3 (2 for BMI more than 40 and 1 for smoking status), heparin prophylaxis was commenced from 28 weeks as per national guidelines. She had a uterine artery Doppler at 24 weeks, which was normal; the risk assessment of her placental function due to smoking was satisfactory. The serial foetal growth scans reported good growth velocity of the fetus along the 50th centile. She was reviewed by the obstetric anaesthetist in view of her raised BMI.

In the third trimester, she continued not to meet her protein requirements and was advised again to aim for dietary protein intake to be not lower than 25% of total energy. She was also advised that her dietary fat should not exceed 25% of total energy to prevent ketosis. She commenced daily fasting and post‐meal capillary blood glucose monitoring from 28 weeks of her gestation, and most readings were reported within normal ranges except for two low values below the target range of 4–10 mmol/L. The patient followed standard advice to address the low blood glucose, which quickly returned within normal range. Her HbA1c was within the normal range.

She remained clinically stable throughout her pregnancy with no muscle pains or weakness, although her CK concentration was raised at 1025 U/L during pregnancy. She continued to take cornstarch late at night and multivitamin supplements.

ALT was 73–77 U/L in each trimester. GGT was within normal ranges throughout the pregnancy. In the first trimester total cholesterol was 6 mmol/L and triglycerides 3 mmol/L.

Remote continuous glucose monitoring (CGMS) monitoring was offered to the patient in the second trimester but the patient chose not to use. She did monitor her blood glucose regularly only 2 brief episodes of hypoglycaemia were reported to the Metabolic team or her obstetrician. She also reported no symptoms of low blood glucose when asked.

## 4 Intrapartum Management

Induction of labour was planned for 37 + 3 weeks gestation with a prostaglandin pessary. Her capillary blood glucose was monitored every 2 h, and she was able to take cornstarch or oral 20% glucose polymers until active labour. She received a stat dose of 100 mg of pethidine with 50 mg of cyclizine intramuscularly for analgesia during the induction process. Foetal tachycardia was noted 7 h post prostaglandin pessary. She had 4+ ketones in urine, and her blood ketones were 0.6. She was very keen on vaginal birth, and once foetal tachycardia was confirmed to be uncomplicated, artificial rupture of membranes was performed with difficulty in view of raised body habitus. Her labour was then augmented with synthetic oxytocin. She was given intravenous dextrose 10% and normal saline throughout the labour.

There was a transient rise in blood pressure, which resolved, and her hourly capillary blood glucose monitoring was normal. She had epidural analgesia (0.1% bupivacaine plus fentanyl 2mcg/ml) in labour, but this appeared to be dislodged. She had a raised temperature of 38.5°C, and septic screen investigations were performed. As per the intrapartum pyrexia in labour protocol, she was administered intravenous paracetamol and broad‐spectrum antibiotics (cefuroxime and metronidazole). Her venous gas pH was normal with a lactate of 1.2 mmol/L. She had reduced urine output at this stage, but her renal function was normal. She progressed to full dilation and had rotational forceps delivery with episiotomy and deep vaginal wall repair in theatre under spinal anaesthesia (2.3 mls of 0.5% bupivacaine and 300mcg of diamorphine). She had a blood loss of 900 mL at the birth of the baby, mainly due to trauma to the perineum.

A baby boy was born at 37 + 3 weeks with a birth weight of 3 kg. Baby required immediate resuscitation at birth with inflation breaths and the baby cried at 4 min of age with umbilical arterial pH of 7.18 and base excess of −9.8 and umbilical venous pH of 7.2 and base excess of −9.3. These results suggest no evidence of intrapartum hypoxia. Apgar scores were 4, 8 and 9 at 1 min, 5 min and 10 min of life, respectively (Apgar score describes the condition of the newborn after birth and takes into account the colour, heart rate, reflexes, muscle tone and respiration of the newborn). The maximum score is 10 and any score more than 7 is reassuring.

Baby was observed for hypoglycaemia in the first 24 h and had a septic screen due to maternal pyrexia in labour. His CRP was raised, and he received intravenous antibiotics for 5 days. Baby required phototherapy for neonatal jaundice.

## 5 Post‐partum Management

She developed transient acute kidney injury (AKI 2) at 24 h post birth with eGFR of 58 and creatinine of 115 mmol/L and was seen by an AKI nurse. An infection and long labour were likely causes of her renal dysfunction. She continued to receive intravenous antibiotics and fluids, and renal function improved 12 h later with good urine output, with full normalisation 48 h post birth (eGFR > 90 and serum creatinine 64umol/L). She discontinued oral antibiotics 5 days afterwards. Her post‐partum haemoglobin was 86 g/L on day 1 post‐partum, with a spontaneous improvement on day 4 (Hb 132 g/L). Post‐partum ALT was 97 U/L and CK was 1463 U/L.

She was reviewed by a renal nurse at 3 months post‐partum and she continued to have normal renal function. She was prescribed Low‐molecular‐weight heparin for 6 weeks for VTE prophylaxis. She was debriefed by her obstetrician about the events surrounding labour, and she declined formal debriefing at 6 weeks post‐partum. Despite minor complications in labour and post‐partum period, overall she had a successful forceps delivery and mother and baby were discharged home on day 6. The baby was formula milk fed.

She reported generalised pains and myalgia after 11 weeks post‐partum. Within the first 12 months after post‐partum she tried a commercial weight loss programme and managed to reduce her body weight. A follow‐up in metabolic clinic showed stable biochemical parameters and no new symptoms.

In view of her underlying cardiomyopathy, she underwent further assessment. Cardiac MRI showed severe focal left ventricular hypertrophy, with maximum wall width measuring 19 mm in the basal anteroseptum (an area of the heart not well visualised on echocardiogram). The left ventricular systolic function was supranormal at 78%. On late gadolinium images, there was the appearance of focal fibrosis in the mid inferior and inferoseptal segments (Suppl Figure 1A,B,C).

Resting 12 lead ECG showed sinus rhythm with lateral Q waves, fractionated QRS complexes, and high lateral T wave inversion, an unusual pattern of ECG abnormality within cardiomyopathy. Ambulatory ECG showed a short run of asymptomatic, slow, non‐sustained ventricular tachycardia.

The genetic mutation analysis was requested as part of whole genome sequencing to correlate her cardiac findings with her genotype. Our patient has been found to be heterozygous for two pathogenic *AGL* variants, c.798C>G p.(Tyr266Ter) and c.4258_4259ins? p.(Asp1420fs) (Supplementary file).

## 6 Discussion

This case report describes a relatively mild form of GSD III in a female patient who had an uncomplicated pregnancy. This patient had fibrotic changes in the myocardium caused by an unusual pathogenic variant.

Although the pregnancy was uncomplicated, the patient developed renal complications and cardiac arrhythmia after delivery. This was thought to be an indirect complication of the pregnancy.

Setner et al. also found that cardiomyopathy was associated with increased distal myopathy and muscle soreness after minimal‐intensity exercise [6]. Our patient clinically manifested generalised myalgia and stable hypertrophic cardiomyopathy and fibrosis.

Apart from the risk of hypoglycaemia, patients with pre‐existing cardiomyopathy could have cardiac complications during pregnancy due to the physiological changes linked to pregnancy [13, 16]. Left ventricular hypertrophy and increased cardiac muscle mass are common findings in GSD IIIA patients, but less so in GSD IIIB patients [17]. Cardiac fibrosis in GSD III has previously been documented on histopathology analysis of the endocardium in post‐mortem cases [18]. The findings from the cardiac MRI of our patient showed mild–moderate focal LVH with focal septal myocardial fibrosis seen on late gadolinium enhancement images, which was in keeping with the previous report [18].

It is crucial to perform periodic checks of ventricular function. Adequate management of fluids, pre‐surgery preparation, pain control, and monitoring after delivery are essential.

Nutritional requirements increase during pregnancy. Pregnant patients with GSD III are advised to have a daily nutritional intake based on a 55% slow‐release carbohydrate, 25% protein, and fat portion below 25% [13, 19]. If energy requirements are not met, there is a risk of hypoglycaemia with subsequent negative consequences for the intrauterine development of the foetus, including intrauterine growth restriction and low birth weight [15]. To prevent hypoglycaemia, cornstarch should be started at a dose of 1 g/kg at bedtime or more if there are symptoms, ranging from 0 to 2.3 g/kg/day, as soon as patients become pregnant.

A high‐protein diet has been shown to have a positive impact on improving muscle symptoms and cardiac manifestations [20]. Due to the lack of strong evidence for specific protein requirements for pregnant GSD III patients, advice on protein intake should also consider the standard recommended for pregnant patients [13]. It is noted that this patient did not achieve a dietary protein intake consistent with recommendations for pregnancy or for patients with GSD III.

Ramachandran et al. have described the largest cohort of pregnant women with GSD III [13]. They report 6 uncomplicated pregnancies in 3 women with GSD IIIA and 9 pregnancies in 4 women with GSD IIIB. There was no consensus regarding the management, with some women receiving only dextrose infusion, some having a combined normal diet and cornstarch, and some others not requiring additional calorie supplementation.

The management in this case was guided by capillary glucose concentration [21]. This patient was managed with dextrose infusion throughout the labour and delivery without hypoglycaemic episodes similar to other cases described in literature [13].

The lactation period has an impact on the metabolic control and basic metabolic rate. Breastfeeding is associated with an increase in calorie intake. Any necessary dietetic modifications should be made respective to the metabolic control. Our patient was not able to breastfeed, and her son was formula fed instead.

Hepatic adenomas may increase in size during pregnancy. Despite close surveillance of her abnormal liver function tests and alpha‐fetoprotein prior to pregnancy, liver adenomas were not observed in this case.

## 7 Conclusions

Careful pre‐emptive planning and multidisciplinary work during pregnancy and the post‐partum period minimise the risk of metabolic and obstetrics complications for the mother and her baby. Women with GSD III are at risk of alterations in the metabolic rate during pregnancy and the post‐partum period. Close management of diet with regular monitoring is necessary to ensure that the risk of hypoglycaemia is minimised. However, this case demonstrates the importance of post‐partum monitoring for patients to fully manage direct and indirect complications of pregnancy in GSD III. Cardiac monitoring during and after pregnancy is essential in women with this condition. This should include monitoring for cardiac disease as well as fibrotic changes in their myocardium.

## Author Contributions

N.P.‐R. and K.M.S. – concept design. N.P.‐R. – literature review and manuscript draft. All authors – data acquisition and the manuscript edition. All the authors approved the final version. K.M.S. – Guarantor.

## Conflicts of Interest

The authors declare no conflicts of interest.

## Supporting information


**Data S1.** Suppl Figure 1A: Cardiac MRI bSSFP sequence 2 chamber image; Severe focal left ventricular hypertrophy of the basal anterior segment.
Suppl Figure 1B: Cardiac MRI bSSFP sequnce short axis image; Severe focal left ventricular hypertrophy of the basal anterior and anteroseptal segment.
Suppl Figure 1C: Cardiac MRI late gadolinium enhancement sequence. Bright focal area of myocardial fibrosis in the mid inferoseptum.
Suppl Figure 2: Detection of the *AGL* c.4258_4259ins? p.(Asp1420fs) variant. (a) manual inspection of the WGS data using IGV for complex variants in the *AGL* gene showed presence of sequence from chr6p12.1 at the exon/intron 31 boundary (indicated as the sequence in boxes within the reads—grey lines indicate a match to AGL sequence). (b) sanger sequence data confirming the insertion of chr 6p12.1 sequence into exon 31 of the *AGL* gene.

## Data Availability

The authors confirm that the data supporting the findings of this study are available within the article [and/or] its Supporting Information.
