# Supplementary material for: The Management and Clinical Outcomes of Pregnancy in a Female With Glycogen Storage Disease Type IIIA Caused by Rare Variant
Source: JIMD Rep. 2025 Jul 2;66(4):e70030. doi: 10.1002/jmd2.70030 (PMC12221335; doi:10.1002/jmd2.70030)
Supplement: Supplementary file 1 — Data S1. Suppl Figure 1A: Cardiac MRI bSSFP sequence 2 chamber image; Severe focal left ventricular hypertrophy of the basal anterior segment. Suppl Figure 1B: Cardiac MRI bSSFP sequnce short axis image; Severe focal left ventricular hypertrophy of the basal anterior and anteroseptal segment. Suppl Figure 1C: Cardiac MRI late gadolinium enhancement sequence. Bright focal area of myocardial fibrosis in the mid inferoseptum. Suppl Figure 2: Detection of the AGL c.4258_4259ins? p.(Asp1420fs) variant. (a) manual inspection of the WGS data using IGV for complex variants in the AGL gene showed presence of sequence from chr6p12.1 at the exon/intron 31 boundary (indicated as the sequence in boxes within the reads—grey lines indicate a match to AGL sequence). (b) sanger sequence data confirming the insertion of chr 6p12.1 sequence into exon 31 of the AGL gene. [file JMD2-66-e70030-s001.docx]

Suppl Fig 1 Cardiac magnetic resonance 12 months after the pregnancy; MRI showed LVH with myocardial fibrosis. A) Cardiac MRI bSSFP sequence 2 chamber image; Severe focal left ventricular hypertrophy of the basal anterior segment. B) Cardiac MRI bSSFP sequnce short axis image; Severe focal left ventricular hypertrophy of the basal anterior and anteroseptal segment. C) Cardiac MRI late gadolinium enhancement sequence. Bright focal area of myocardial fibrosis in the mid inferoseptum.

A. B.


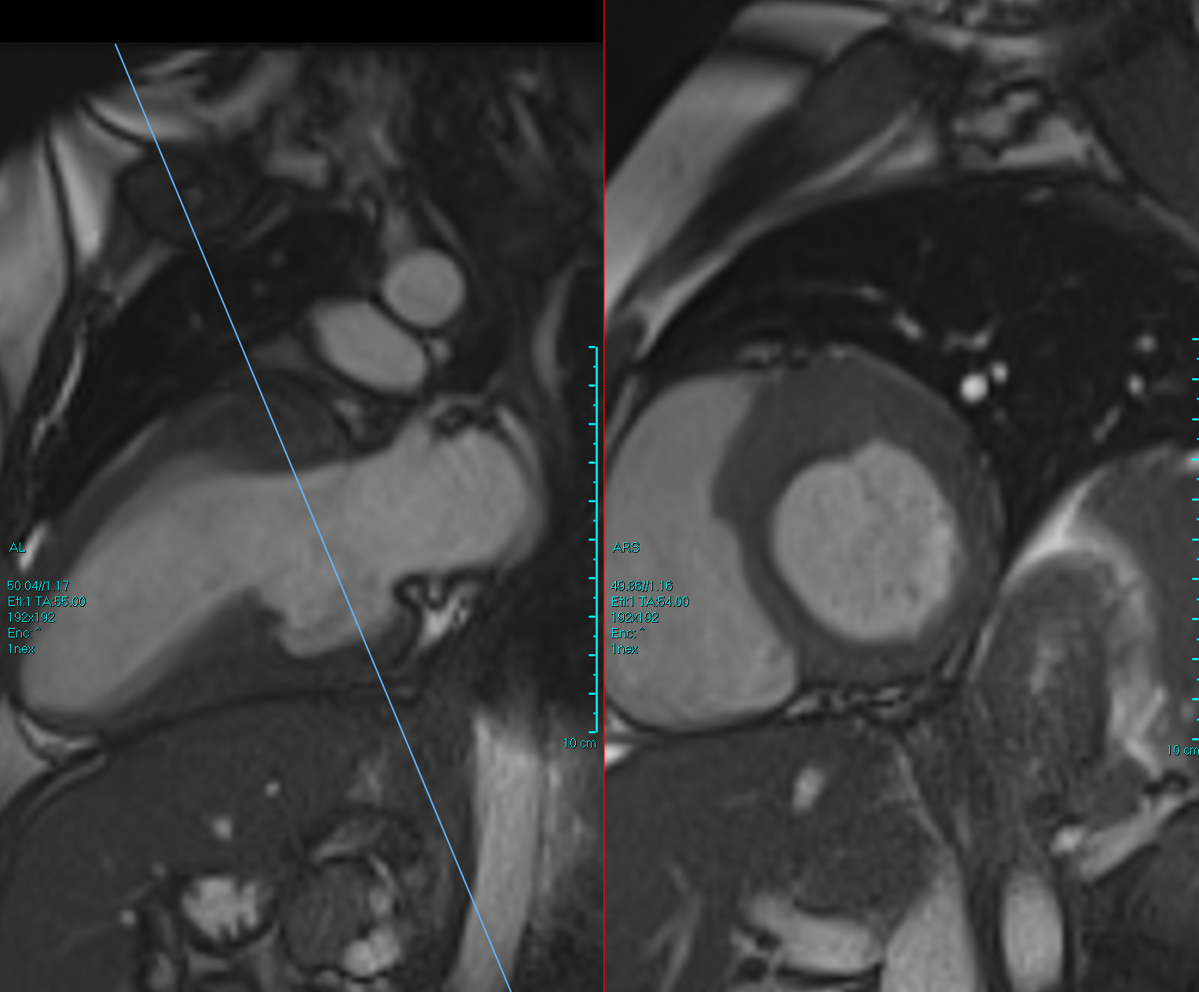


Left ventricle

Left atrium

18mm

18mm

Interventricular septum

Left ventricle

Right ventricle

C.


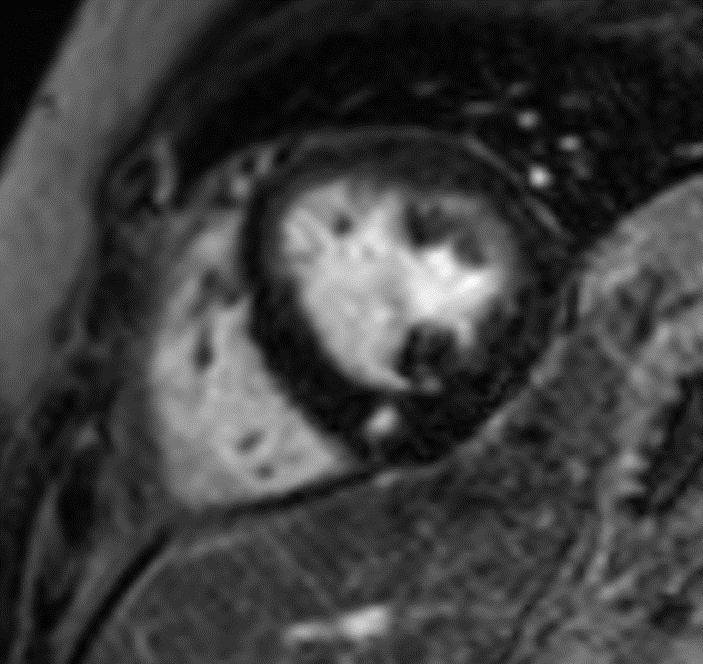


Left ventricle

Right ventricle

Myocardial fibrosis

Normal myocardium

A)

Figure 2

Short axis late gadolinium enhancement image:

Myocardial enhancement indicating fibrosis in the hypertrophied inferior septum

Suppl Figure 2. Detection of the AGL c.4258_4259ins? p.(Asp1420fs) variant. A) manual inspection of the WGS data using IGV for complex variants in the *AGL* gene showed presence of sequence from chr6p12.1 at the exon/intron 31 boundary (indicated as the sequence in boxes within the reads – grey lines indicate a match to AGL sequence). B) sanger sequence data confirming the insertion of chr 6p12.1 sequence into exon 31 of the *AGL* gene.

A.


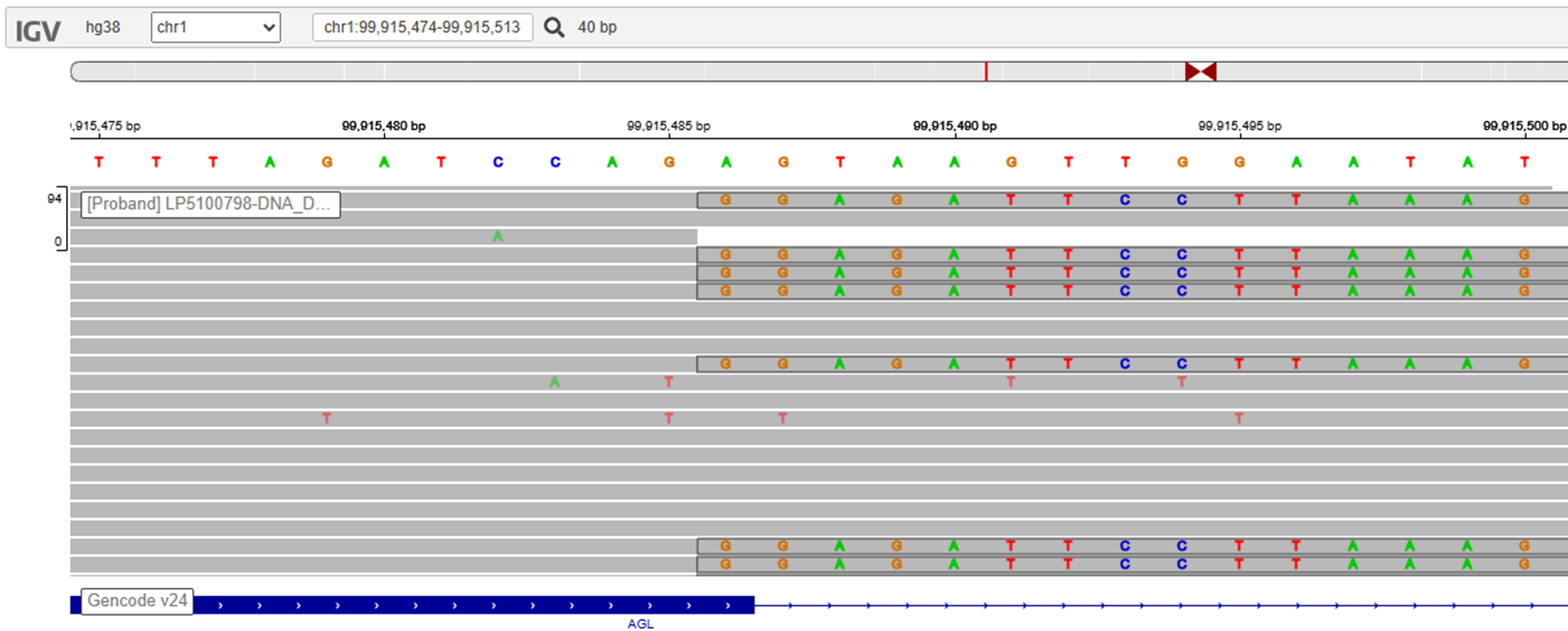


Our patient has been found to be heterozygous for two pathogenic *AGL* variants, c.798C>G p.(Tyr266Ter) and c.4258_4259ins? p.(Asp1420fs). Both of these variants result in a premature termination codon and are predicted to be subject to nonsense mediated decay and AGL loss of function is a well-established mechanism of disease for GSD type III^1,2^.

Stringent filtering of Next Generation Sequencing (NGS) and Whole Genome Sequencing (WGS) data filters out the *AGL* c.4258_4259ins? p.(Asp1420fs) variant and it was detected by manual inspection of the data due to the known diagnosis of GSD III in the patient. Misaligned reads were observed at position c.4258 which is the exon/intron 31 boundary of the *AGL* gene (NM_000642), representing an insertion of retrotransposed DNA (of undetermined size) from the short arm of chromosome 6 band p12.1 (see Suppl Figure 2A and 2B). A forward primer located in the *AGL* gene in chromosome 1 and a reverse primer located in chromosome 6 were used to amplify the junction region and sanger sequencing of this 148bp fragment showed the presence of a sequence that is identical with a segment in chromosome 6 (6p12.1) confirming a genomic rearrangement involving the *AGL* gene on chromosome 1 (1p21.2) and chromosome 6 (6p12.1) (see Suppl Figure 2B,). Sequence analysis cannot determine the size of this insertion from chromosome 6, however it is predicted to result in a frameshift within the *AGL* gene resulting in a null allele.

The genomic rearrangement involving 1p21.2 and 6p12.1 (Suppl Figure 2), which disrupts the *AGL* gene, has been previously reported in a study where ‘loose’ filtering and alignment conditions were used for thorough analysis of misaligned NGS reads^3^. It has also been detected in another UK patient via manually inspection of the WGS data (information provided by the East Genomic Laboratory Hub) and confirmation by sanger sequencing across the junction fragment as outlined above. The patient does not have frequent symptomatic hypoglycaemia but takes Glycosade at bedtime and at 1am at night.

As the *AGL* c.4258_4259ins? p.(Asp1420fs) pathogenic variant has now been reported in 3 patients with GSD III and is not detected by standard bioinformatic pipelines used for NGS and WGS analysis, screening for this variant should be considered for patients with a clinical diagnosis of GSD III where the genetic cause has not been confirmed.

1. OMIM Entry – # 232400 – Glycogen Storage Disease III; GSD3″. www.omim.org. Retrieved 2024-08-11.
2. Goldstein JL, Austin SL, Boyette K, Kanaly A, Veerapandiyan A, Rehder C, Kishnani PS, Bali DS. Molecular analysis of the AGL gene: identification of 25 novel mutations and evidence of genetic heterogeneity in patients with Glycogen Storage Disease Type III. *Genet Med*  2010;12(7):424-30.
3. Wang J, Yu H, Zhang VW, Tian X, Feng Y, Wang G, Gorman E, Wang H, Lutz RE, Schmitt ES, Peacock S, Wong L-J. Capture-based high-coverage NGS: a powerful tool to uncover a wide spectrum of mutation types. *Genet Med* 2016 May;18(5):513-21.).

Suppl Figure 2B.

B)


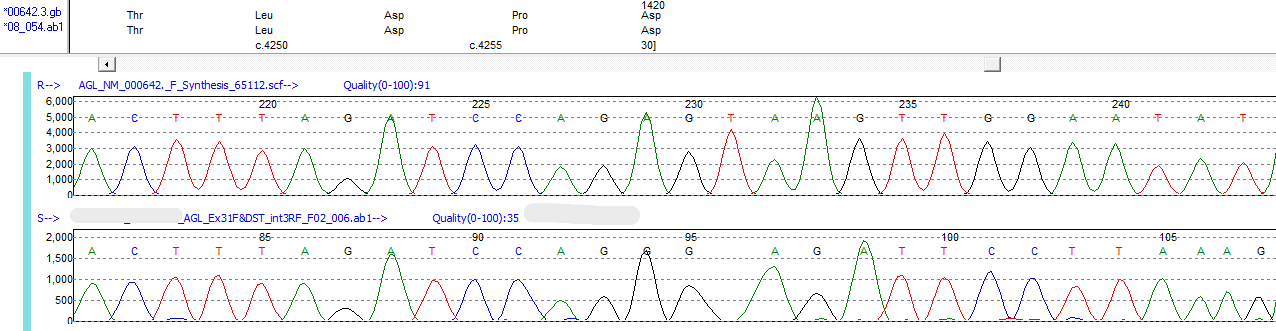


Patient DNA sequence

AGL exon/intron 31 reference sequence: NM_000642
